# Epidemiological Analysis of Displaced Supracondylar Fractures

**DOI:** 10.7759/cureus.7734

**Published:** 2020-04-19

**Authors:** Nick I Pilla, John Rinaldi, Mark Hatch, William Hennrikus

**Affiliations:** 1 Orthopaedics, Penn State College of Medicine, Penn State Milton S. Hershey Medical Center, Hershey, USA; 2 Orthopaedics, Allegheny General Hospital, Pittsburgh, USA; 3 Orthopaedics, Rosenberg Cooley Metcalf Orthopedic Clinic, Park City, USA; 4 Orthopaedic Surgery, Penn State Health Milton S. Hershey Medical Center, Hershey, USA

**Keywords:** supracondylar, pediatric fractures, paediatric orthopedics, pediatric trauma, upper extremity

## Abstract

Introduction: Supracondylar fractures are one of the most common fracture patterns sustained by children, and one of the most common injuries requiring operative fixation. Understanding the complications associated with supracondylar fractures is vital for the practicing orthopedic surgeon. This analysis of supracondylar fractures examined the clinically important aspects including vascular injury, compartment syndrome, neurological injury, brachialis entrapment, associated injuries, and etiologies of injury. Recent advances in technology have resulted in a myriad of new forms of recreational equipment for children to play with. The purpose of this study is to compare the historical literature, the current literature, and a single surgeon’s sample of supracondylar fractures. In addition, this study aims to evaluate if any changes in epidemiology or etiology have occurred due to the development of new recreational equipment.

Objective: The purpose of this study is to evaluate and provide a qualitative overview of the epidemiology of displaced supracondylar fractures, to compare historically reported numbers to more recent literature as well as a single surgeon sample, and to evaluate if changes in epidemiology or etiology have occurred due to the new recreational equipment that children use.

Methods: Some 75 displaced supracondylar elbow fractures were reviewed. Data elements recorded from the electronic medical record (EMR) included patient age, gender, height, weight, handedness, date, time, location, mechanism, Gartland classification, concurrent injuries, and neurovascular status.

Results: In this study, there were 42 males and 33 females. The average age was six years. Some 70 of the 75 patients were older than the age three. One fracture was open, nine fractures had a pucker sign, seven presented with a nerve palsy, four presented without a pulse, and seven patients presented with an additional ipsilateral distal radius fracture. All fractures were the result of a fall. Falls from playground equipment resulted in 29 fractures. There were 10 from falls off of furniture, six from falls during sports, three from falls on the stairs, and three from fall off of bikes. The remaining fractures resulted from running, tripping, falling from a toy ball, sled, tree, wagon, fence, bounce house, van, deck, power wheels car, ATV, and a go-cart.

Some 64 fractures were transferred from 27 different outside hospitals. Eleven fractures presented directly to the ED. Twenty-six fractures occurred during the summer, 20 occurred in the autumn, 6 occurred in the winter, and 23 occurred during the spring. Some 35 fractures occurred at home, 30 on the school grounds, four in a gymnasium, four in a park, one at a farm show, and one in a parking lot. Some 25 fractures were treated between midnight and 8 am, 16 were treated between 8 am and 5 pm, and 34 were treated between 5 pm and midnight.

Conclusion: Pediatric supracondylar fractures are common in children, and many of them require operative intervention. This study examined the most important aspects of supracondylar fractures. This update provides a look at the clinically important aspects of supracondylar fractures and compares them to previous teachings and canon. Despite the advancement and changes in recreational equipment that children are using, children are still sustaining supracondylar fractures in the most common ways including falls from playground equipment and falls from standing.

## Introduction

Supracondylar fractures are the most common elbow fracture in the pediatric population. Supracondylar fractures comprise 60% of all elbow fractures [1]. This qualitative analysis of supracondylar fractures examined the clinically important aspects of supracondylar fractures including vascular injury, compartment syndrome, neurological injury, brachialis entrapment, associated injuries, etiologies of injury, and indications for transfer. There have been many new forms of recreational equipment developed for children to play with over the years. Some of these new forms of recreational equipment include hoverboards, electric scooters, and motorized toy vehicles for children to ride in. The purpose of this study is to compare the historical literature, the current literature, and a single surgeon’s sample of supracondylar fractures, as well as to evaluate if any changes in epidemiology or etiology have occurred due to the new recreational equipment that children use.

## Materials and methods

The College of Medicine Institutional Review Board (IRB) has approved this study. The electronic medical records (EMRs) and radiographs for all patients who underwent operative fixation of supracondylar humeral fractures by the senior author (W.H.) from 2010 to 2014 were analyzed. Seventy-five consecutive displaced supracondylar elbow fractures were reviewed.

Data elements recorded from the EMR included patient age, gender, height, weight, handedness, date, time, location, mechanism, Gartland classification of presenting fracture, date and time of transfer and/or ED arrival, and physical exam findings including associated fractures, concurrent injuries, and neurovascular status. Results of the current study were compared to the historical canon.

## Results

Forty-two males (56%) and 33 females (44%) were studied. The average age was six years (range: one year four months to 12 years four months). Two patients were one-two years of age, three were two-­three years of age, and 70 were older than three. The left elbow was fractured in 45 cases (60%). Some 87% were right hand dominant, 9% were left hand dominant, and 4% were too young to determine handedness. Some 58% injured the non­dominant arm. Twenty-three (31%) were Gartland Type 2 and 52 (69%) were Gartland Type 3 fractures. 

One fracture was open. Nine (12%) fractures had a pucker sign. Seven (9%) presented with a nerve palsy. Four (5%) presented without a pulse. Seven patients (9%) presented with an additional ipsilateral distal radius fracture. Sixty-four fractures (85%) were transferred from 27 different outside hospitals in 17 counties, while 11 fractures (15%) presented directly to the ED. Twenty-six (35%) fractures occurred during the summer, 20 (27%) in the autumn, 6 (8%) in the winter, and 23 (31%) during the spring season. Thirty-five fractures (47%) occurred at home, 30 (40%) on the school grounds, four (5%) in a gymnasium, four (5%) in a park, one at a farm show, and one in a parking lot. Twenty-five of the fractures were treated between midnight and 8 am, 16 were treated between 8 am and 5 pm, and 34 were treated between 5 pm and midnight.

**Figure 1:**
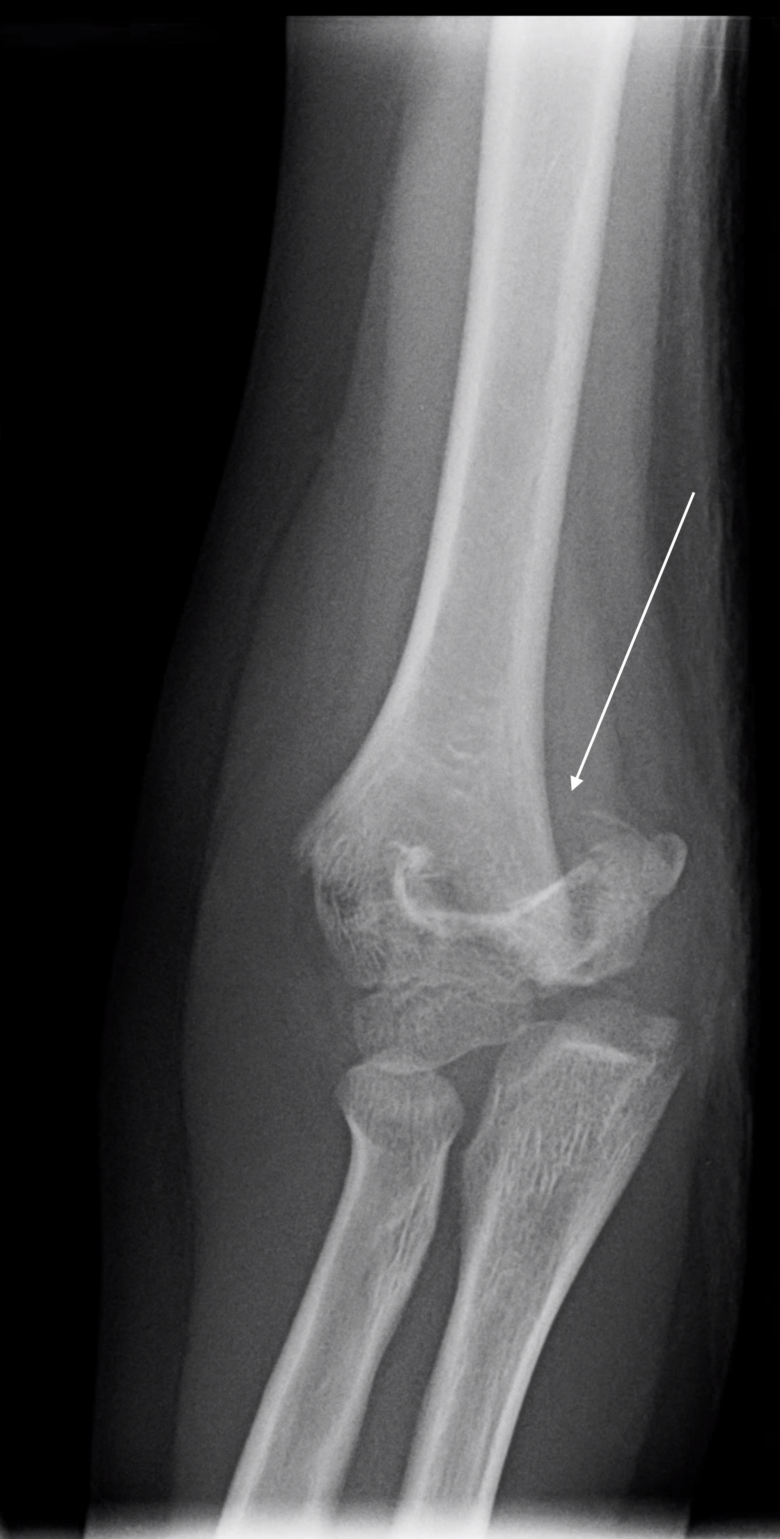
AP view of a Type 2 supracondylar fracture in seven-year-old girl who fell off of a trampoline. AP, anteroposterior

**Figure 2:**
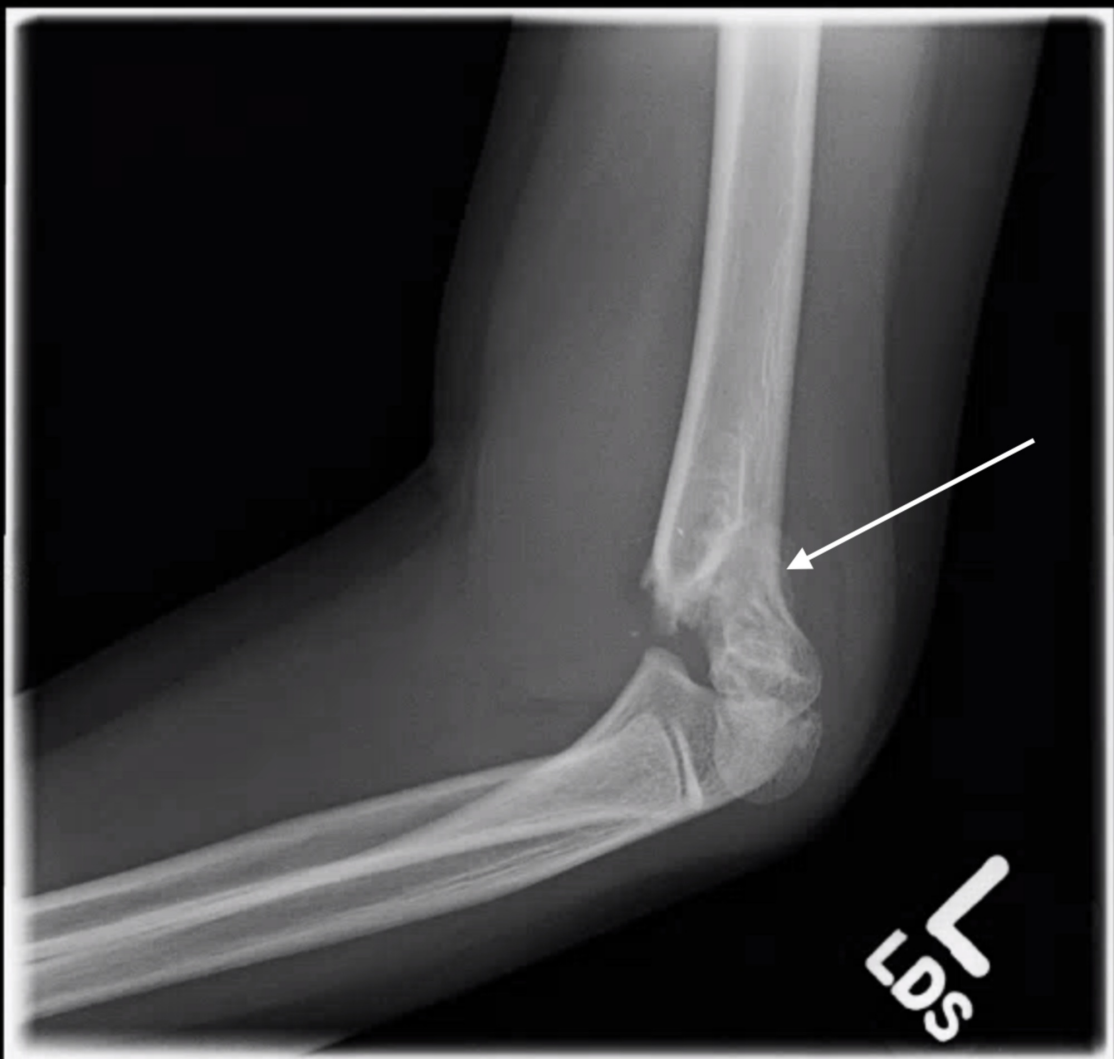
Lateral view of a Type 2 supracondylar fracture in a seven-year-old girl who fell off of a trampoline.

**Figure 3:**
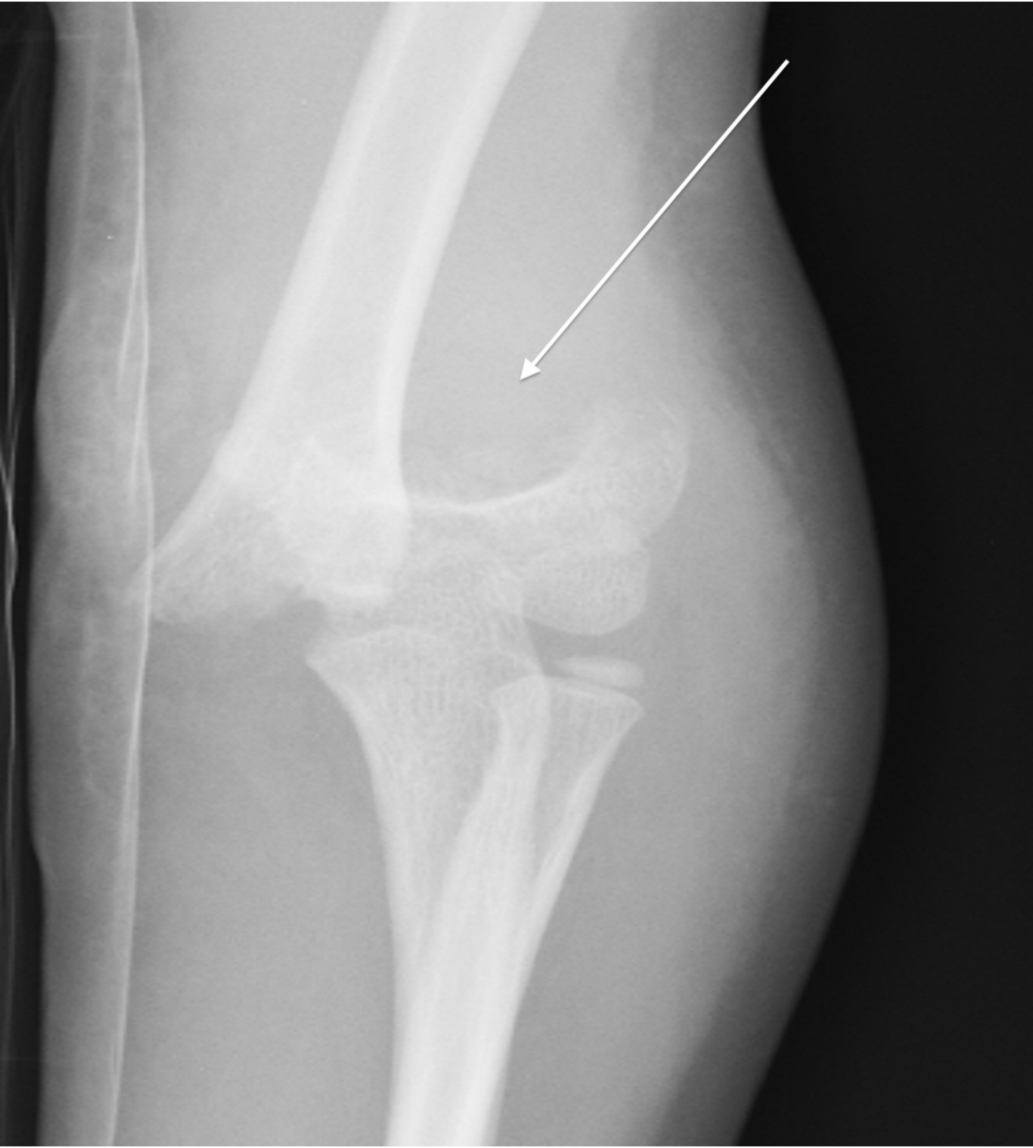
AP view of a Type 3 supracondylar fracture in a six-year-old boy after a fall from the monkey bars. AP, anteroposterior

**Figure 4:**
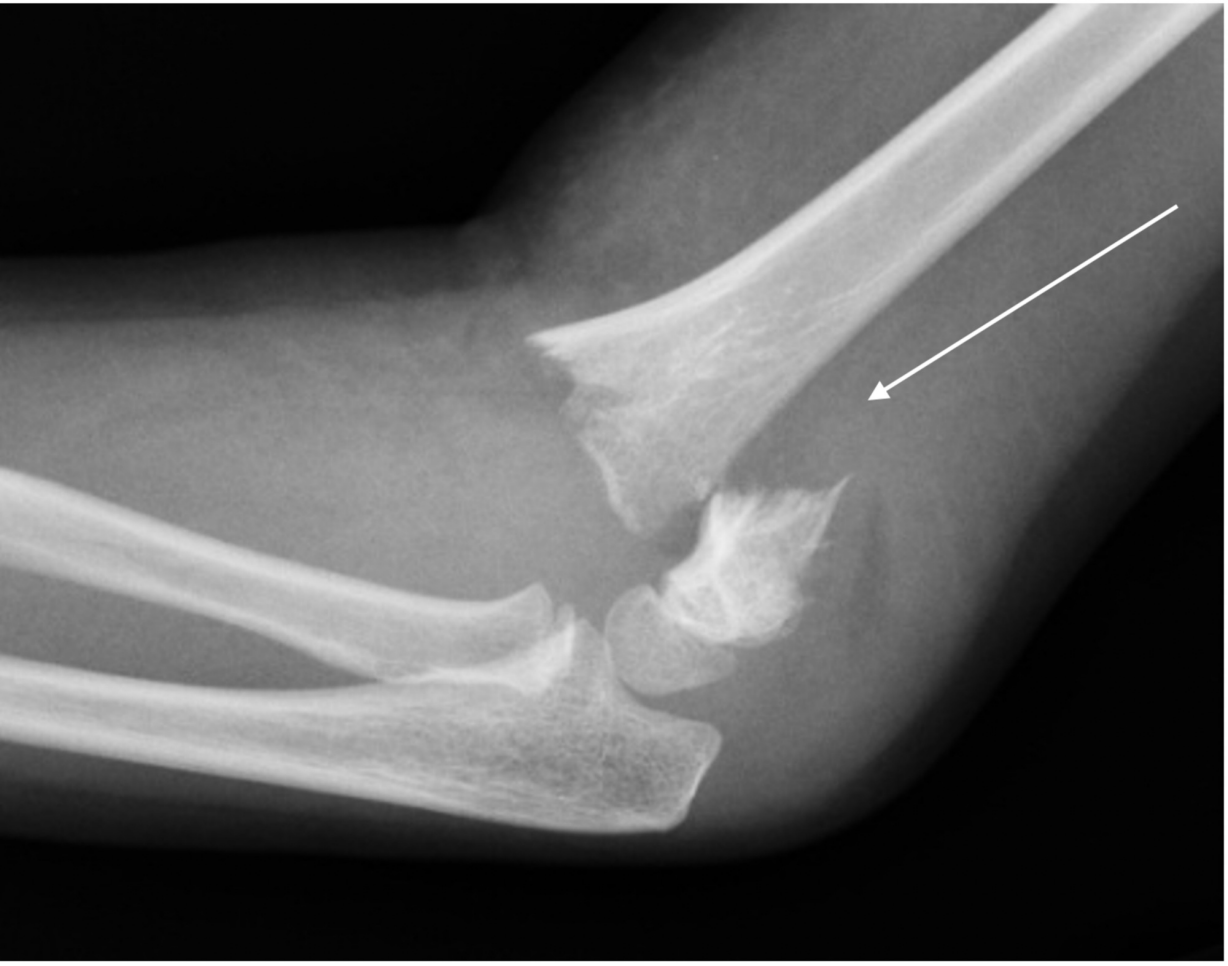
Lateral view of a Type 3 supracondylar fracture in a six-year-old boy after a fall from the monkey bars.

All fractures stemmed from a reported accidental fall, including: 29 (39%) from playground equipment, 10 (13%) from furniture, six (8%) while playing sports, three (4%) from falling down stairs, and three (4%) from riding bicycles. The remaining 24 (32%) patients fell performing miscellaneous activities including: running and tripping, falls from a toy ball, sled, tree, wagon, fence, bounce house, mini-van, deck, battery-powered ride-in car, all-terrain vehicle (ATV), and a go-cart. Tables 1-5 demonstrate the demographic data, fracture breakdown, mode of arrival vs. fracture type, and mode of arrival vs. transfer type. Figures 1-4 represent examples of two children included in the study that sustained a Gartland Type 2 and Gartland Type 3 supracondylar fractures respectively. 

 

**Table 1:** General patient characteristics for children with supracondylar fractures.

Patient characteristics	
Male	42 (56.0%)
Female	33 (44.0%)
Avg. age at time of fracture	6 years, 2 months
Right-hand dominant	65 (86.7%)
Left-hand dominant	7 (9.3%)
Handedness not documented	3 (4.0%)
Avg. Height (cm)	115.5
Avg. Weight (kg)	22.7

**Table 2:** General supracondylar fracture data.

Fracture data	
# of years	4 (2010-2014)
Supracondylar fracture, total	75
Gartland Type II	23 (31%)
Gartland Type III	52 (69%)

**Table 3:** Supracondylar associated complications.

Presenting complications	
Associated fractures, total	12
# of patients with associated fractures	7 (9%)
Compartment syndrome	0 (0%)
Nerve injury	7 (9%)
Vascular injury	4 (5%)
Pucker sign	9 (12%)

**Table 4:** Mode of arrival vs. Gartland Type.

Mode of arrival	Type 2	Type 3
Transferred (n=64)	19 (30%)	45 (70%)
Private automobile	16	17
Ambulance	3	26
Helicopter	0	2

**Table 5:** Mode of arrival vs. Admit Type.

Mode of arrival	All	Transferred	Direct admit to Hershey Medical Center
	75	64	11
Private automobile	40 (53%)	33 (52%)	7 (64%)
Ambulance	33 (44%)	29 (45%)	4 (34%)
Helicopter	2 (2.6%)	2 (3%)	0 (0%)

## Discussion

Age

The average age was six years old. Cheng et al., in 1999, reported on 1,162 children with supracondylar fractures. Some 43.5% (505) of the fractures occurred in children between the ages of four and seven [2]. In a second article in 2001, Cheng et al. examined 403 children with supracondylar fractures and found the average to be six years old [3]. In the current study, the average age was six years old. 

Gender prevalence 

Historically, males have had a higher incidence of supracondylar humeral fractures [3]. On the other hand, in 1999, Farnsworth et al. reported on 391 fractures and concluded that there were more fractures in girls than boys in the zero-one and six-seven age groups [4]. They reported that there were no significant differences between boys and girls in all other age groups. Mehlman et al. recently reported a 2:1 ratio in children under two [5]. Farnsworth and Mehlman both reported that the female incidence is higher in children less than two. One possible explanation is that the humeral width and area are thinner in prepubertal girls than prepubertal boys [6]. In the current study, only two patients were aged less than two; one was male, and one was female. Some 56% of the patients were male. 

Mechanism of injury 

The most common mechanism reported is a fall on an outstretched hand. Children are susceptible to supracondylar fractures due to a thinning of the distal humeral cortex [7]. Furthermore, the olecranon fossa in conjunction with ligamentous laxity in the pediatric elbow allows for hyperextension of the elbow [7]. 

In four separate studies totaling 1,671 supracondylar fractures, falls in general from all sources made up 1,442 (86%). Specifically, playground equipment related injuries and falls accounted for 329 (20%) of the fractures. Monkey bar falls are the most common cause of playground-related supracondylar fracture. In the same four studies totaling 1,671 supracondylar fractures, 144 were the direct result of falls from monkey bars (7.5%) [4, 8-11]. Another common culprit resulting in supracondylar fractures are trampoline falls. In three studies totaling 1,237 supracondylar fractures, 128 were the result of a fall from a trampoline (10%) [8-10]. Monkey bars and trampolines alone account for between 15% and 20% of supracondylar fractures. Other common mechanisms reported include falling off of furniture, falling from a standing height, falling while playing, and many other falls from miscellaneous activities that children engage in. 

In the current study of 75 supracondylar fractures, all 75 stemmed from a reported fall, including 29/75 (39%) from playground equipment, 10/75 (13%) from furniture, 6/75 (8%) while playing sports, 3/75 (4%) from falling down-stairs, and 3/75 (4%) from riding bicycles. The remaining 24/75 (32%) patients fell performing miscellaneous activities, including: running and tripping, falls from a toy ball, sled, tree, wagon, fence, bounce house, mini-van, deck, battery-powered ride-in car, ATV, and riding a go-cart. The site of the fall was varied. 35/75 (47%) occurred at the child’s home, 30/75 (40%) on school grounds, 4/75 (5.3%) in a gymnasium, 4/75 (5.3%) in a park, 1/75 (1.2%) at a farm show, and 1/75 (1.2%) in a parking lot. 

The American Academy of Pediatrics (AAP) has gone so far as to recommend against the use of trampolines for children to play on stating that pediatricians need to actively discourage recreational trampoline usage [11]. The findings of the current study indicate that the AAP should focus on monkey bar safety as well. 

Vascular injury 

Vascular complications secondary to a supracondylar fracture can lead to compartment syndrome and subsequent ischemic contracture. This study defined vascular injury as a diminished or lack of distal radial pulse upon presentation. Studies evaluated included only displaced supracondylar fractures. 

In a review of seven studies, totaling 3,468 supracondylar fractures, 259 (6.7%) presented with a nonpalpable pulse [4, 12-17]. The range of supracondylar fractures presenting with vascular injury has been reported as few as 2.6% and as many as 17%. In the current study of 75 patients with supracondylar fractures, four (5.3%) presented with a vascular injury. 

Although rare, Choi et al. reported that 44% of supracondylar fractures with a pulseless limb and clinical signs of poor perfusion will require a vascular repair. Choi reviewed 1,255 displaced supracondylar fractures treated operatively. Some 33/1,255 (2.6%) presented with a pulseless hand. Of those 33, 24 had a well-perfused hand, and nine had a poorly perfused hand. Of the 24 with a well-perfused hand, zero required vascular repair to restore a radial pulse, three required open reduction, and the other 21 required closed reduction with percutaneous pinning. Of those 21, 11 had a palpable pulse after surgery, 10 did not but had a well-perfused hand; all did well clinically. Of the nine patients with a poorly perfused hand upon initial presentation, 4/9 required vascular injury repair, and of those, two of them developed compartment syndrome. The other five only required fracture reduction to restore vascularity [17]. Overall, 4/1,255 fractures (0.003) required vascular repair. 

Neurological injury 

Nerve injury has been reported as the most common complication after supracondylar fractures. The injury is usually a neuropraxia. Most cases resolve with up to six months of observation. In some cases, it can be difficult to tell whether the nerve injury was a result of the trauma or the treatment. The incidences of traumatic nerve injuries have previously been reported between 12% and 35% while the incidence of iatrogenic nerve injuries has been reported between 2% and 6% [18-19]. Injury to the median/anterior interosseous nerve (AIN) and radial nerve are a result of extension-type supracondylar fractures. Ulnar nerve injuries are usually to flexion type injuries or iatrogenic percutaneous pinning of the fracture. 

In a review of two studies, from 2010 and 2017, including the largest meta-analysis of its kind by Babal et al., 5,266 supracondylar fractures were evaluated and 648 nerve injuries (12%) occurred. The nerve injuries included 205 AIN injuries, making it the most common nerve-injured (32%). Some 135 (20%) suffered median nerve injuries, 10 (1.5%) suffered posterior interosseous nerve (PIN) injuries, 173 (27%) suffered radial nerve injuries, and 125 (19.5%) suffered ulnar nerve injuries [19, 20]. In the current study of 75 patients with supracondylar fractures, seven patients suffered a nerve injury (9%). Nerve injuries included seven AIN and one ulnar. All nerve palsies resolved with observation. Individuals who sustain a nerve palsy with a congruent supracondylar fracture demonstrate poorer outcomes with regard to pain, function, mobility, and satisfaction at final follow-up despite complete resolution without any type of surgical or procedural intervention [18, 21].

Brachialis entrapment/pucker sign 

The brachialis or pucker sign indicates that the fracture has penetrated through the brachialis muscle. The result is a puckering of the skin in the supracondylar region. The presence of the pucker sign has been suggested to be an indicator of difficulty in reducing the fracture. The pucker sign is associated with potential neurovascular compromise. Two studies totaling to 195 supracondylar fractures were evaluated. Of those, 28 (14%) presented with brachialis entrapment [22-23]. In the current study of 75 patients with supracondylar fractures, nine of them had a pucker sign (12%). The current recommendation for the treatment of a fracture with a pucker sign includes gradual traction and milking the soft tissue until reduction is achieved [24]. 

Additional fractures 

Most supracondylar fractures are isolated injuries; some are associated with other fractures. Most often they are the result of low impact activities such as a fall from height. In a review of three studies totaling 1,071 supracondylar fractures, a total of 97 additional fractures (9%) were reported [3- 4, 12]. In these three studies, the most common additional fracture was an ipsilateral forearm fracture which was reported as the additional fracture in 52 of the 97 additional fractures (53%). In the current study of 75 patients, there were 87 total fractures. Of the 75 children with supracondylar fractures, 12/75 (16%) had an additional fracture. The most common additional fracture sustained was an ipsilateral distal radius or ulna fracture reported in 12/12 fractures (100%). 

Season 

A review of two studies in addition to this study was evaluated with regard to the effect of seasons on supracondylar fractures. Six-hundred-ninety-six fractures from three different climates were evaluated including Toronto, San Diego, and Pennsylvania [4, 12]. Sixty-six percent occurred in the spring and summer (March-September). Supracondylar fractures occur more frequently in the spring and summer months regardless of geographic location. 

Compartment syndrome 

Compartment syndrome of the forearm is a rare, but potentially devastating complication associated with supracondylar fractures. Muscle tissue and nerve ischemia are the major risk risks of missing compartment syndrome, resulting in tissue death and compartment contracture [25]. 

In a review of two studies totaling 2,069 supracondylar fractures, there were five (0.2%) cases of compartment syndrome in isolated supracondylar fractures [17, 26]. This is consistent with the national data provided by the Robertson et al.: 67 isolated supracondylar fractures resulted in compartment syndrome out of the national data of 31,167 supracondylar fractures (0.2%) [26]. Of note, several studies including the study by Blakemore et al. examined an additional 43 children with supracondylar fracture and ipsilateral forearm fractures. Of those, three developed compartment syndromes. This study indicates that additional fractures associated with a supracondylar fracture increase the risk for compartment syndrome [27]. In the current study of 75 patients with supracondylar fractures, there were no reported cases of compartment syndrome. 

In addition to the fracture the treatment of the fracture can lead to compartment syndrome. Preventative measures such as avoiding more than 90 degrees of flexion, uni-valving, or bi-valving the cast may release upwards of 77% of the pressure within a cast [28]. 

Indications for transfer

According to the ACGME requirements, diagnosis and treatment of a pediatric supracondylar fracture is an orthopedic residency core competency. A list of these requirements can be found on the ACGME website [29]. Every graduating orthopedic resident has met the supracondylar fracture requirements competency, thereby demonstrating expertise in managing this injury. Despite meeting competency requirements, the majority of supracondylar fractures continued to be transferred. In the current study as many as 85% of supracondylar fractures were transferred to our center. 

One reason for which transfer of a supracondylar fracture should occur is if no orthopedic surgeon is available to care for fracture. A second reason is if a hand remains pulseless or dysvascular following reduction, and the center does not have a plastic or vascular surgeon available. This scenario occurs in less than 1% of cases.

Delays in treatment due to transfers are common. Delays in treatment can lead to an increase in morbidity to patients and their families including compartment syndrome requiring fasciotomy [30]. In this single surgeon study of 75 supracondylar fractures, 85% of them were transferred from outside facilities. In the current study, no compartment syndromes occurred. 

About 12% of children with supracondylar fractures may present with a neurological palsy. Almost all of these nerve injuries recover with six months of observation. A nerve palsy on its own is not an indication for transfer. 

Limitations

Limitations of the study include use of a single surgeon’s sample as a reference, a homogenous cohort of subjects (80% Caucasian), the retrospective design, and analysis of all the studies involved.

## Conclusions

Pediatric supracondylar fractures are the most common surgically treated fracture in children. This study examined the most important facets pertaining to supracondylar fractures including age, gender prevalence, mechanism of injury, vascular injury, neurological injury, brachialis sign, presence of additional fractures, seasonal preference, compartment syndrome, and indications for transfer. This update provides a look at some of the most important aspects of supracondylar fractures and compares them to previous teachings and canon. Despite the advances and changes in recreational equipment, children are still sustaining supracondylar fractures in the classical ways including playground equipment and falls.

## References

[1] Lins RE, Simovitch RW, Waters PM (1999). Pediatric elbow trauma. Orthop Clin North Am.

[2] Cheng JC, Ng BK, Ying SY (1999). A 10-year study of the changes in the pattern and treatment of 6,493 fractures. J Pediatric Orthop.

[3] Cheng JC, Lam TP, Maffulli N (2001). Epidemiological features of supracondylar fractures of the humerus in Chinese children. J Pediatric Orthop B.

[4] Farnsworth CL, Silva PD, Mubarak Mubarak, SJ SJ (1998). Etiology of supracondylar humerus fractures. J Pediatric Orthop.

[5] Mehlman CT, Denning JR, McCarthy JJ (2019). Infantile supracondylar humeral fractures (patients less than two years of age). J Bone Joint Surg.

[6] Clark EM, Ness AR, Tobias JH (2006). Gender differences in the ratio between humerus width and length are established prior to puberty. Osteop Int.

[7] Nork SE, Hennrikus WL, Loncarich DP (1999). Relationship between ligamentous laxity and the site of upper extremity fractures in children. J Pediatric Orthop B.

[8] Mitchelson AJ, Illingoworth KD, Robinson BS (2013). Patient demographics and risk factors in pediatric distal humeral supracondylar fractures. Orthopedics.

[9] Barr LV (2014). Paediatric supracondylar humeral fractures: epidemiology, mechanisms and incidence during school holidays. J Children’s Orthop.

[10] Siriwardhane M, Siriwardhane J, Lam L (2018). Supracondylar fracture of the humerus in children: mechanism of injury. Orthop Proc.

[11] Brenner JS, Benjamin HJ, Cappetta CT (2012). Trampoline safety in childhood and adolescence. Off J Am Acad Pediatrics.

[12] Pirone AM, Graham HK, Krajbich JI (1988). Management of displaced extension-type supracondylar fractures of the humerus in children. J Bone Joint Surg.

[13] Shaw BA, Kasser JR, Emans JB (1990). Management of vascular injuries in displaced supracondylar humerus fractures without arteriography. J Orthop Trauma.

[14] Luria S, Sucar A, Eylon S (2007). Vascular complications of supracondylar humeral fractures in children. J Pediatr Orthop B.

[15] Louahem D, Cottalorda J (2016). Acute ischemia and pink pulseless hand in 68 of 404 gartland type III supracondylar humeral fractures in children: Urgent management and therapeutic consensus. Injury.

[16] Ho CA, Podeszwa DA, Riccio AI (2018). Soft tissue injury severity is associated with neurovascular injury in pediatric supracondylar humerus fractures. J Pediatr Orthop.

[17] Choi PD, Melikian R, Skaggs DL (2010). Risk factors for vascular repair and compartment syndrome in the pulseless supracondylar humerus fracture in children. J Pediatr Orthop.

[18] Ramachandran M, Birch R, Eastwood DM (2006). Clinical outcome of nerve injuries associated with supracondylar fractures of the humerus in children: the experience of a specialist referral centre. J Bone Joint Surg Br.

[19] Babal JC, Mehlman CT, Klein G (2010). Nerve injuries associated with pediatric supracondylar humeral fractures: a meta-analysis. J Pediatr Orthop.

[20] Gera SK, Tan M, Lim YG (2017). Displaced supracondylar humerus fractures in children - are they all identical?. Malays Orthop J.

[21] Ernat JJ, Riccio AI, Wimberly RL (2015). Nerve injury predicts functional outcomes in pediatric supracondylar humerus fractures: a prospective study. J Hand Surg.

[22] Peters CL, Scott SM, Stevens PM (1995). Closed reduction and percutaneous pinning of displaced supracondylar humerus fractures in children: description of a new closed reduction technique for fractures with brachialis muscle entrapment. J Orthop Trauma.

[23] Archibeck MJ, Scott SM, Peters CL (1997). Brachialis muscle entrapment in displaced supracondylar humerus fractures: a technique of closed reduction and report of initial results. J Pediatr Orthop.

[24] Smuin DM, Hennrikus WL (2017). The effect of the pucker sign on outcomes of type III extension supracondylar fractures in children. J Pediatr Orthop.

[25] Bae DS, Kadiyala RK, Waters PM (2001). Acute compartment syndrome in children: contemporary diagnosis, treatment, and outcome. J Pediatr Orthop.

[26] Robertson AK, Snow E, Browne TS (2018). Who gets compartment syndrome?: a retrospective analysis of the national and local incidence of compartment syndrome in patients with supracondylar humerus fractures. J Pediatr Orthop.

[27] Blakemore LC, Cooperman DR, Thompson GH (2000). Compartment syndrome in ipsilateral humerus and forearm fractures in children. Clin Orthop Relat Res.

[28] Zaino CJ, Patel MR, Arief MS (2015). The effectiveness of bivalving, cast spreading, and webril cutting to reduce cast pressure in a fiberglass short arm cast. J Bone Joint Surg Am.

[29] (2020). Accreditation Council for Graduate Medical Education. https://www.acgme.org/Specialties/Milestones/pfcatid/14/Orthopaedic.

[30] Ramachandran M, Skaggs DL, Crawford HA (2008). Delaying treatment of supracondylar fractures in children: has the pendulum swung too far?. J Bone Joint Surg Br.

